# Screening for Methylmalonic and Propionic Acidemia: Clinical Outcomes and Follow-Up Recommendations

**DOI:** 10.3390/ijns8010013

**Published:** 2022-02-07

**Authors:** Patrice K. Held, Emily Singh, Jessica Scott Schwoerer

**Affiliations:** 1 Department of Pediatrics, University of Wisconsin School of Medicine and Public Health, Madison, WI 53706, USA; jscottschwoerer@gmail.com; 2 Wisconsin State Laboratory of Hygiene, University of Wisconsin School of Medicine and Public Health, Madison, WI 53706, USA; 3 Division of Genetics, Medical College of Wisconsin with Children’s Wisconsin, Milwaukee, WI 53226, USA; esingh@mcw.edu

**Keywords:** newborn screening, methylmalonic acidemia, propionic acidemia, methylmalonic acid, methylcitric acid, homocysteine, second-tier testing, liquid chromatography tandem mass spectrometry

## Abstract

Wisconsin’s newborn screening program implemented second-tier testing on specimens with elevated propionylcarnitine (C3) to aid in the identification of newborns with propionic and methylmalonic acidemias. The differential diagnosis for elevated C3 also includes acquired vitamin B12 deficiency, which is currently categorized as a false positive screen. The goal of this study was to summarize screening data and evaluate their effectiveness at establishing diagnoses and categorizing false positive cases. All Wisconsin newborns born between 2013 and 2019 with a positive first-tier screen for C3 were included in this study. For each case the first- and second-tier newborn screening data and confirmatory test results were compiled. The clinical determination for each case was reviewed and categorized into groups: inborn error of metabolism, maternal B12 deficiency, infant B12 deficiency, and false positive. A review of the screening data showed a significant overlap in the concentration of biomarkers for newborns with genetic versus acquired disease. Additionally, a review of confirmatory test results showed incomplete ascertainment of maternal vitamin B12 status. The Wisconsin newborn screening program recommended a confirmatory testing algorithm to aid in the diagnosis of inborn errors of metabolism and acquired vitamin B12 deficiency.

## 1. Introduction

Newborn screening is a public health program aimed at improving outcomes for children with treatable disorders that are not detected by routine care. Many of the disorders appropriate for newborn screening are inborn errors of metabolism (IEM), including propionic acidemia (PA), methylmalonic acidemia, and disorders of cobalamin metabolism. Propionic acidemia (OMIM #606054) and methylmalonic acidemia (OMIM #251000) are due to enzyme deficiencies in propionyl CoA carboxylase and methylmalonic CoA mutase, respectively, and can present in the newborn period with acidosis and hyperammonemia or later in life with metabolic decompensations and/or end organ damage including cardiomyopathy and renal disease [1,2]. Disorders of cobalamin metabolism, including cobalamin C (cblC) deficiency (OMIM #277400), are associated with the activation of cobalamin to be used in enzymatic pathways. The phenotype for disorders of cobalamin metabolism is variable but includes global developmental delay, encephalopathy, neurologic symptoms, and cytopenia [3]. It has been well documented that newborn screening for methylmalonic acidemia and PA improves outcomes, and more recent studies also show improved outcomes for cblC deficiency [1,2,3,4,5,6,7].

Wisconsin introduced expanded newborn screening by tandem mass spectrometry in early 2000, which allows for the identification of infants with PA, methylmalonic acidemia, and disorders of cobalamin metabolism using propionylcarnitine (C3) as the disease biomarker. The screening algorithm was later modified to include the ratio of propionylcarnitine to acetylcarnitine (C3/C2) as a primary biomarker. In 2013, Wisconsin lowered the cutoff value for C3 from <6.92 µM to <5.0 µM and implemented second-tier testing for methylmalonic acid (MMA) and methylcitric acid (MCA) to maximize the sensitivity and specificity of the screen, as shown by other published studies [8,9,10,11,12,13]. It is widely accepted that early ascertainment of MMA, MCA, and total homocysteine levels through second-tier testing aids in classification of the disease and its severity. While the value of total homocysteine has been demonstrated in recent studies, it is not included in the second-tier assay for abnormal C3 newborn screens in Wisconsin.

Similar to methylmalonic acidemia and disorders of cobalamin metabolism, infant and maternal vitamin B12 deficiency leads to increased concentrations of both C3 and MMA and can be detected on the newborn screen [12,13,14]. In fact, pilot studies performed in several countries using these markers have indicated an incidence of vitamin B12 deficiency between 1 in 5355 and 1 in 80,000 [8,9,12,13,14,15]. At present, vitamin B12 deficiency is not part of newborn screening panels in the United States. Currently, acquired vitamin B12 deficiency is often detected during the first year of life, with nonspecific symptoms including developmental delay, failure to gain weight, irritability, hypotonia, difficult weaning, and anemia. Long-term sequelae of sustained vitamin B12 deficiency may include a poor intellectual outcome due to irreversible brain damage [16,17,18,19,20,21]. Infants most at risk of vitamin B12 deficiency are those who are breastfeeding and whose mothers have additional risk factors for vitamin B12 deficiency, including malabsorption and dietary restrictions [16,17,18,19,20,21]. Given that both infant and maternal vitamin B12 deficiency can be identified as a result of elevated C3 on the newborn screen and poses long-term risks to infant health and development, we propose that it may be necessary to evaluate mothers as well as their infants for vitamin B12 deficiency as part of the confirmatory testing process to ensure accurate diagnosis and intervention.

As with all abnormal screening results, confirmatory testing is imperative to confirm the presence of disease; however, there is no consensus on which confirmatory tests should be performed. The American College of Genetics and Genomics’ algorithm for the evaluation of isolated elevated C3 recommends urine organic acids, plasma acylcarnitine, and total homocysteine (HCY) [22]. GeneReviews recommends a more extensive panel of tests for both the newborn and mother, including urine organic acids, plasma acylcarnitine, plasma amino acids, HCY, serum MMA, and serum vitamin B12 in the newborn, as well as serum vitamin B12 for the mother [2]. A prior study of individuals with vitamin B12 deficiency showed high sensitivity for serum MMA and HCY as markers of tissue vitamin B12 status [23]. A number of reports describe vitamin B12-deficient infants, as determined by clinical sequelae, maternal vitamin B12 status, and/or laboratory values, with elevated MMA and HCY despite normal or low-normal vitamin B12 levels [12,24,25]. As such, although serum vitamin B12 is currently recommended for confirmatory testing, the combination of MMA and HCY may be a better marker of maternal and neonatal vitamin B12 deficiency [26].

The intended goal of this quality improvement study is to summarize diagnostic outcomes in newborns with elevated C3 on the newborn screen as well as evaluate confirmatory testing practices across two metabolic centers. This study recognizes the need for consistent clinical follow-up and provides a recommended confirmatory testing algorithm with added emphasis on the evaluation for vitamin B12 deficiency.

## 2. Materials and Methods

### 2.1. Specimens

From 1 January 2013 through 31 December 2019, the Wisconsin newborn screening program referred 176 of the 458,139 screened newborns for medical genetics consultation and confirmatory testing due to an increased risk for propionic acidemia (PA) or methylmalonic acidemia. All 176 cases had elevated propionylcarnitine (C3) and/or ratio of propionylcarnitine to acetylcarnitine (C3/C2) on the initial screening specimen collected between 24 and 72 h after birth. At the time of data analysis, confirmatory testing results and assessment of clinical outcome was still pending for 3 of the 176 newborns. A total of 6 newborns from the group of 176 were lost to follow-up, and the cases were closed based on normal second-tier testing. As a quality improvement study, the newborn screen data and confirmatory test results for the remaining 167 newborns were reviewed and re-categorized by the collaborative team of PH, JSS, and ES, representing the facilities WSLH, UW, and MCW, respectively. All data are summarized as an aggregate with no identifiable information.

### 2.2. First- and Second-Tier Assays

Wisconsin uses a laboratory developed, non-derivatized flow injection analysis coupled with tandem mass spectrometry assay for quantification of acylcarnitines in dried blood spots. Briefly, a 1/8” punch of each dried blood spot specimen is placed into a 96-well plate, and the biomarkers are extracted with 100 µL of methanol containing labeled acylcarnitine species as internal standards. The plate is incubated at room temperature, shaking, for 10 min, diluted with methanol, then loaded onto an autosampler where 10 µL of the sample is injected into a stream of mobile phase (80:20 acetonitrile–water with 0.1% formic acid). The AB Sciex API 4000 or API 4500 tandem mass spectrometers with a TurboV electrospray ionization source are operated in positive mode with three looped experiments. In the first experiment, acylcarnitines are measured by precursor ion scanning and 85 amu fragment detection. In the second and third experiments, amino acids are measured by a constant neutral loss scan of 46 amu or 63 amu. Detection and measurement of the acylcarnitines is accomplished using Analyst 2.0.3 with a companion ChemoView 2.0.6 software (Sciex, Framingham, MA, USA).

The second-tier assay designed to quantitate methylmalonic acid (MMA) and methylcitric acid (MCA) in dried blood spots has been modified slightly from previous publications [27]. In the Wisconsin method, MMA and MCA are extracted from a 3/16” punch of the dried blood spot specimen with 150 µL of a 70:30 acetonitrile–water solution containing labeled internal standards for each acid. The plate is incubated, shaking, for 15 min at room temperature and then loaded onto an autosampler. Separation of the two acids is performed using a Gemini 3µ C6-Phenyl 110 Å 100 × 2.0mm column (Phenomenex) coupled to an AB Sciex API 4500 tandem mass spectrometer with a TurboV electrospray ionization source operating in negative mode, using multiple reaction monitoring for detection. Two mobile phases of water with 0.5% formic acid and acetonitrile with 0.5% formic acid are used in combination to elute each acid from the column, and adequate baseline separation is achieved with a run time of eight minutes. An external calibration curve prepared from dried blood spots spiked with known concentrations is used to quantify each acid within Analyst software.

Wisconsin performs the second-tier assay two to three times per week. On average, there are five specimens requiring second-tier analysis on each run. Because of the quick run times for the first- and second-tier assays, and the non-derivatized assessment of analytes, results for specimens with elevated propionylcarnitine can typically be reported within 24 to 36 h.

### 2.3. Wisconsin Screening Algorithm

**Figure 1:**
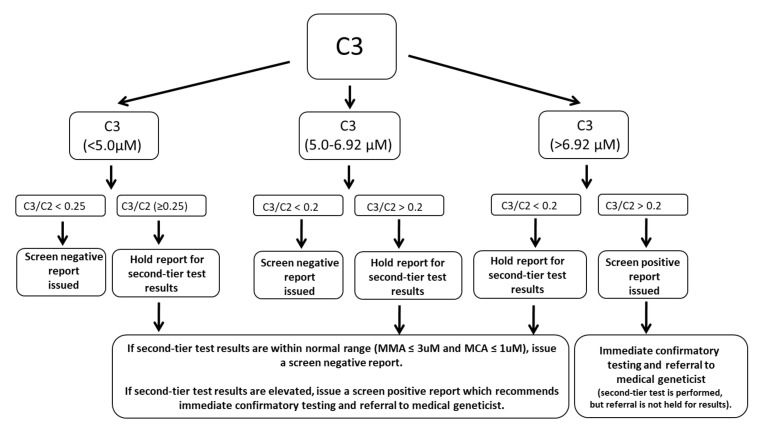
Wisconsin newborn screening algorithm for elevated propionylcarnitine (C3).

The Wisconsin screening algorithm incorporates second-tier testing of MMA and MCA when first-tier markers, C3 and/or C3/C2 ratio, are elevated. The algorithm is designed to identify core conditions on the Recommended Uniform Screening Panel (RUSP), including PA, methylmalonic acidemia due mutase deficiency, and methylmalonic acidemia due to cobalamin disorders (cblA, cblB). Total homocysteine is not included in the second-tier assay as identification of secondary conditions on the RUSP, including MMA with homocystinuria (cblC), is not the primary goal of the newborn screening program. The algorithm, shown in Figure 1, outlines two potential screening outcomes: screen negative results with no further action required or screen positive results that recommend confirmatory testing and consultation with a medical geneticist. Newborns with markedly elevated C3 and/or C3/C2 ratio are referred immediately for consultation and confirmatory testing, prior to acquisition of second-tier test results. Specimens with mild elevations of C3 and/or C3/C2 ratio are held until second-tier results are complete, and only newborns with elevated MMA and/or MCA are referred for consultation and confirmatory testing.

### 2.4. Confirmatory Testing and Metabolic Evaluation Protocol

In Wisconsin, medical genetics clinics are located at Children’s Wisconsin/Medical College of Wisconsin (MCW) in Milwaukee and the Waisman Center at the University of Wisconsin—Madison (UW) in Madison. Newborns with screen positive test results are referred to either clinic based on proximity to their home. Both clinics recommend urine organic acids and plasma acylcarnitines in newborns as part of the confirmatory testing. In addition, MCW recommends serum vitamin B12, and UW recommends plasma homocysteine, serum MMA, and plasma carnitine. As part of the differential diagnosis to exclude maternal vitamin B12 deficiency, MCW recommends serum vitamin B12 in mothers, and UW recommends urine organic acids, plasma homocysteine, serum MMA, and plasma carnitine in mothers. Molecular testing was completed when confirmatory test results raised concern for a true inborn error of metabolism. All testing is completed by commercial referral laboratories according to CLIA-compliant protocols.

Both clinics recommend vitamin B12 supplementation for infants with elevated methylmalonic acid levels, either as measured on second-tier or confirmatory testing. Practices varied on whether 1 mg of oral cyanocobalamin daily or a weekly injection of 1 mg hydroxocobalamin was started while the diagnostic workup was being performed. In either case, vitamin B12 supplementation was continued until methylmalonic acid levels normalized. Methylmalonic acid levels were checked one, three, and six months after discontinuation of supplementation to assure levels remained normal, which was considered consistent with transient maternal or infant vitamin B12 deficiency rather than an inborn error of metabolism.

## 3. Results

### 3.1. Review and Classification of Cases

Between 1 January 2013 and 31 December 2019, the Wisconsin newborn screening program tested 458,139 newborns for propionic acidemia (PA) and methylmalonic acidemia. The first-tier assay identified 1685 newborn specimens with elevated propionylcarnitine (C3) and/or ratio of propionylcarnitine to acetylcarnitine (C3/C2), who then underwent second-tier testing for quantification of methylmalonic acid (MMA) and methylcitric acid (MCA). From this cohort, 167 newborns received medical genetics consultation and confirmatory testing based on the elevated C3 and/or C3/C2 ratio by first-tier analysis or a elevated MMA and/or MCA by second-tier analysis.

An inborn error of metabolism was identified in 14 of the 167 newborns (Table 1). Eight newborns, all members of the Amish community in Wisconsin, were identified with either PA (5 newborns) or cobalamin C deficiency (three newborns) by targeted variant analysis. All eight newborns shared common founder mutations characteristic of each disease. One additional newborn, which was not part of the Amish community, was identified with cobalamin C deficiency based on a biochemical and molecular assessment of *MMACHC*. Methylmalonic acidemia was identified in 5 of the 14 newborns. All five cases had mild presentations, with only minimal elevations of serum MMA (<25 µM). Molecular testing identified two pathogenic variants in *MMUT* in four newborns. One newborn with methylmalonic acidemia had two variants in the transcobalamin receptor gene (*CD320*) identified by the gene panel (Table 2). Based on analysis of this cohort, the positive predictive value (PPV) of the screening for propionic acidemia (PA) and methylmalonic acidemia increased by almost 10%, from 0.83% (14/1685) to 8.4% (14/167), with the implementation of the second-tier assay.

The remaining 153 cases were labeled as false positives for PA, methylmalonic acidemia, or a cobalamin disorder. The study team of PH, JSS, and ES reviewed the available newborn and maternal confirmatory test results and clinical diagnostic summaries and created three additional classifications for this cohort: maternal vitamin B12 deficiency, infant vitamin B12 deficiency, and normal infant and maternal testing. Clear maternal vitamin B12 deficiency was documented in 32 out of 153 cases showing elevations of maternal serum MMA (6 cases), low maternal vitamin B12 (12 cases), or both elevated MMA and low vitamin B12 (14 cases). The cause of maternal vitamin B12 deficiency was not investigated as part of this study. A total of 22 out of 153 cases were categorized as infant vitamin B12 deficiency, with clear evidence of elevated MMA in serum and/or urine (21 cases) or elevated C3 on the plasma acylcarnitine profile (1 case). Vitamin B12 supplementation was started if MMA was elevated in infants in either category. The elevated MMA in each newborn was resolved with supplementation of vitamin B12, and there was no evidence of an acquired maternal vitamin B12 deficiency. The third category of false positive cases, normal infant and maternal testing, contained 19 newborns with no evidence of infant or maternal vitamin B12 deficiency. An additional category contained the remaining false positive cases (80 newborns) with incomplete maternal confirmatory testing for which the source of vitamin B12 deficiency could not be identified; of these cases, 32 newborns showed evidence of vitamin B12 deficiency and were treated with vitamin B12 supplementation, and 48 newborns had normal confirmatory test results (Table 1). Other factors leading to false positive such as hyperbilirubinemia were not reviewed.

### 3.2. Review of Laboratory Testing Data

**Figure 2:**
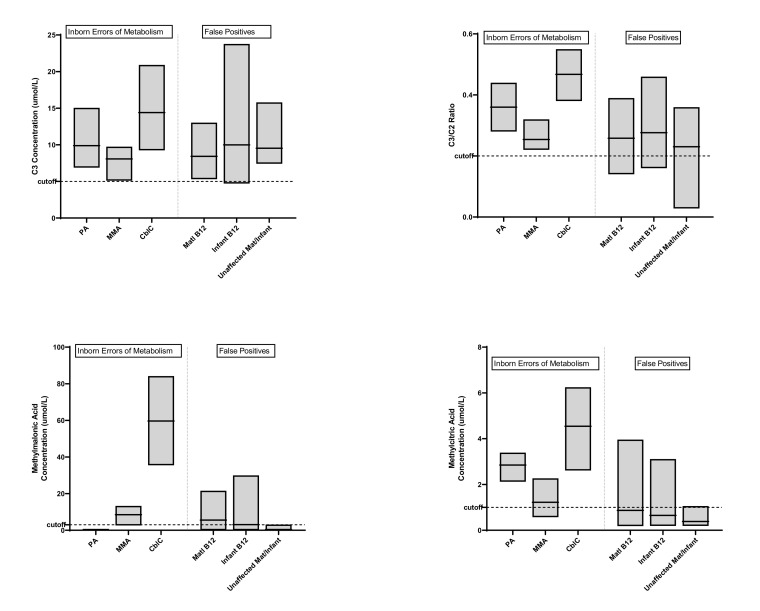
Concentration of disease markers propionylcarnitine (C3), propionylcarnitine to acetylcarnitine ratio (C3/C2), methylmalonic acid, and methylcitric acid in positive C3 newborn screens in Wisconsin from 2013 to 2019. Cases are categorized by final diagnosis of inborn error of metabolism (propionic acidemia (PA), methylmalonic acidemia (MMA), or cobalamin C disease (CblC)) or false positive (maternal vitamin B12 deficiency [matl B12], infant vitamin B12 deficiency, or no evidence for vitamin B12 deficiency (unaffected mat/infant)).

The newborn screening laboratory assessed whether modifications to the cutoff values for either first- and second-tier biomarkers could further improve the sensitivity and specificity of screening (Figure 2, Table 2). In evaluating propionylcarnitine (C3), the mean concentration was similar amongst newborns with propionic acidemia (PA), methylmalonic acidemia, and all false positive cases, while newborns with cobalamin C (cblC) deficiency had an overall higher mean C3 concentration. The range in C3 concentrations was widest in newborns with acquired vitamin B12 deficiency. For all newborns with inborn errors of metabolism, the propionylcarnitine to acetylcarnitine (C3/C2) ratio values were above the cutoff of 0.2, although often close to the threshold, particularly in newborns with methylmalonic acidemia. The concentration of methylmalonic acid (MMA), measured with the second-tier assay, was most distinctly elevated in cases of cblC deficiency. While the mean MMA concentration in methylmalonic acidemia was higher than false positives cases, the ranges overlapped significantly. Methylcitric acid (MCA), also measured by second-tier analysis, was only helpful in distinguishing cases of PA and cblC deficiency from the other groups. In review of additional analytes and ratios (C3/Methionine, C3/C16, C3/free carnitine, and C16:1OH), the only other informative marker for CblC deficiency was the C3 to methionine ratio (data not shown). Changes to the cutoff values for the various analytes and ratios would be unlikely to increase specificity and may in fact lead to decreased sensitivity.

### 3.3. Design of a Clinical Confirmatory Testing Protocol

**Figure 3:**
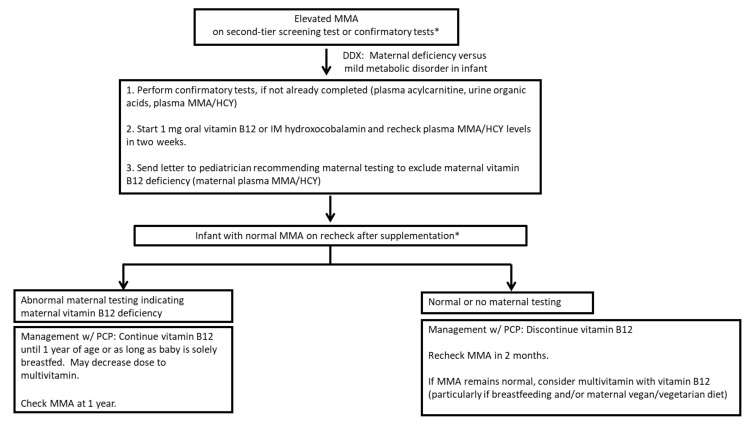
Diagnostic algorithm for evaluation of elevated methylmalonic acid (MMA) on second-tier or confirmatory testing. * Newborns with more abnormalities on confirmatory testing or continued elevation of MMA will be evaluated in a genetics clinic for an inborn error of metabolism.

An evaluation of the confirmatory testing data obtained by the two metabolic centers showed inconsistent practices in the recommended confirmatory testing and variation in the assessment of acquired B12 deficiency. This study developed a unified confirmatory testing algorithm to efficiently screen for an inborn error of metabolism (IEM) as well as evaluate for possible infant or maternal vitamin B12 deficiency (Figure 3). Previously, maternal testing was requested for cases of elevated C3, regardless of second-tier test results, but this was not completed in 80 out of the 153 cases labeled as false positives for PA and methylmalonic acidemia, preventing an accurate evaluation of acquired vitamin B12 deficiency. Based on published evidence that MMA and homocysteine represent better markers for vitamin B12 deficiency, the protocol recommends maternal evaluation only for infants with elevated MMA on second-tier screens.

The algorithm also outlines a schedule for checking MMA levels after vitamin B12 supplementation has been discontinued. This enables a mild vitamin-B12-responsive IEM to be ruled out. The algorithm also recommends the infant’s primary care provider review factors that may put the infant at risk for continued vitamin B12 deficiency, including exclusive breastfeeding and/or the maternal diet. Two versions of educational letters were created, one for the primary care physician and one for the parent, to highlight the importance of evaluating for and treating infant and maternal vitamin B12 deficiency.

## 4. Discussion

It is now commonly accepted that a tiered testing strategy is indispensable for newborn screening programs aimed at identifying disorders of propionate metabolism. In Wisconsin, implementation of second-tier testing to reduce false positive results for disorders of propionate metabolism dramatically improved the positive predictive value of the screen by greater than 10-fold, which is similar to the testing accuracy achieved by other screening programs [28]. The long-term benefits of second-tier testing include programmatic cost savings, higher accuracy in screening, and reduced time to diagnosis [29].

While striving to minimize false positives, newborn screening laboratories must also prevent false negatives. Implementation of the second-tier assay allowed Wisconsin to lower its threshold for propionylcarnitine (C3) to ensure adequate detection of all newborns, as false negative cases have been identified within our program and discussed in the literature [30,31,32]. Indeed, 2 of the 14 patients confirmed to have an inborn error of metabolism (IEM) in our cohort had a C3 level that would have been interpreted as normal if the addition of second-tier testing had not permitted, lowering the C3 threshold. To our knowledge, no additional false negative cases were reported to the metabolic specialists at either of the two centers in Wisconsin during the study’s time frame. Even lower thresholds for C3 were proposed in the recent paper by Pajares et al. to ensure identification of all IEM, making second tier testing all the more crucial to avoid an increase in false positive screens [33].

Typically, screening algorithms for disorders of propionate metabolism are based upon elevated C3 values as the primary marker for identifying a newborn that needs additional evaluation. In our data, there was a wide, overlapping range of C3 concentrations in both genetic and acquired vitamin B12 deficiency cases, with some of the highest values found in newborns determined to be unaffected. Two recent papers substantiate our finding that C3 is not a specific marker for disorders of propionate metabolism [13,33]. Within our data, there was also significant overlap in the C3/C2 values between IEM and false positive cases, which differed from the findings of other groups, who have suggested that C3/C2 provides better sensitivity and is more frequently high in genetic rather than acquired conditions [33,34]. Our data also show that the methylmalonic acid (MMA) concentration obtained on the second-tier test does not help to distinguish an IEM from acquired disease. As such, after reviewing the seven years of data, no additional changes to newborn screening cutoffs could be made to further reduce false positives or sort out genetic from acquired disease.

This highlights the importance of the medical genetics’ evaluation and consistent clinical confirmatory testing practices. This study revealed challenges with the short-term follow-up of positive C3 newborn screens. The two metabolic centers in Wisconsin recommended differing workups for cases with similar screening results. This, in part, stemmed from the lack of clear guidelines for the evaluation of newborn and maternal vitamin B12 status. Often, serum vitamin B12 was recommended, but the literature indicates that MMA and homocysteine represent better markers for vitamin B12 deficiency. In addition, maternal labs were not consistently recommended, and when requested, they were only completed in an estimated 50% of cases, leading to inconsistencies in the evaluation and diagnosis between the metabolic centers in Wisconsin. Therefore, it was difficult to attribute some false positive newborn screens to maternal vitamin B12 deficiency. While the recognition of true IEMs was not impacted, these inconsistencies led to different degrees of short-term clinical intervention and diagnostic outcomes between the metabolic centers for infants ultimately determined to have a false positive newborn screen.

The analysis of the data shows that acquired vitamin B12 deficiency accounts for a significant proportion of false positives—at least 40% (54/135) of abnormal newborn screens with elevated C3 were due to either maternal or infant vitamin B12 deficiency. Sustained vitamin B12 deficiency in infancy can present with non-specific symptoms and lead to long-term, irreversible neurologic sequelae. A review of the literature indicates that infants found to have vitamin B12 deficiency are nearly always breastfed and have a mother with vitamin B12 deficiency due to dietary factors or underlying disease leading to malabsorption. Therefore, a prompt and accurate assessment and diagnosis of vitamin B12 deficiency in the infant or mother is necessary not only to eliminate the concern for an IEM in the setting of an abnormal newborn screen but also to recognize when treatment is needed. Supplementation of vitamin B12 and careful follow-up to ensure the deficiency is resolved for both newborn and mother is important to prevent neurological impacts and improve long-term clinical outcomes, particularly in breastfed infants.

To that end, one product of this study was the design of an algorithm to standardize and improve the short-term follow-up of a positive C3 newborn screen, particularly to evaluate vitamin B12 deficiency as a potential cause of a false positive. With the correct diagnosis, both mother and infant can be treated for deficiency, and monitoring can be completed to ensure adequate treatment. Recognizing that maternal testing can be challenging to obtain in our healthcare system, maternal testing was targeted only to those whose infants had elevated MMA on second-tier or confirmatory test results. With a goal to increase the number of maternal evaluations completed, educational letters for the primary care provider and parents were developed for both Wisconsin metabolic centers. The letters discuss the importance of testing and treatment for vitamin B12 deficiency, as well as the possible long-term neurologic impact on the infant if left untreated. A future study of adherence to this algorithm and its impact on the diagnosis of vitamin B12 deficiency for a positive C3 screen will help further ascertain the number of false positive screens due to infant or maternal vitamin B12 deficiency. It can also help to gauge how increased educational effort may help to complete clinical confirmatory testing and the short-term follow-up in a timely manner.

This study highlights that vitamin B12 deficiency is prevalent, as is consistent with other studies. Clearly, the timing and emphasis of screening for vitamin B12 deficiency is variable. Newborn screening for vitamin B12 deficiency is being considered in the United States and other countries [35]. It may also be feasible to screen mothers during prenatal care. Alternatively, screening for vitamin B12 deficiency could be incorporated into routine child care, particularly for breastfed infants.

## 5. Conclusions

In conclusion, this study recognized that no changes could be made to the current laboratory cut-offs for primary and secondary markers of propionate metabolism to reduce false positives or differentiate disease states. Therefore, the Wisconsin newborn screening program recommended changes to the clinical confirmatory testing algorithm to aid in the diagnosis of inborn errors of metabolism and acquired vitamin B12 deficiency, including maternal deficiency. The algorithm includes an initiative to increase parental and provider education and awareness about maternal deficiencies that can impact a newborn’s health.

## Figures and Tables

**Table 1:** Diagnoses from evaluation of positive propionylcarnitine (C3) newborn screens in Wisconsin from 2013 to 2019.

Inborn Errors of Metabolism	14
Propionic acidemia	5
Methylmalonic acidemia	5
Cobalamin C deficiency	4
**False Positives—Complete Maternal Testing**	**73**
Maternal vitamin B12 deficiency *B12 status described by elevated MMA (6 cases), low B12 (12 cases), elevated MMA + low B12 (14 cases)*	32
Infant vitamin B12 deficiency *B12 status described by elevated serum MMA +/− urine MMA (21 cases), elevated propionylcarnitine (1 case)*	22
No abnormalities detected in infant and maternal testing	19
**False Positives—Incomplete Maternal Testing**	**80**
Infant vitamin B12 deficiency, no maternal testing performed *B12 status described by abnormal serum MMA +/− urine MMA (28 cases), low B12 (2 cases), elevated C3 (2 cases)*	32
No abnormalities detected in infant testing, no maternal testing performed	48
**Total**	**167**

**Table 2:** Inborn errors of metabolism found on propionylcarnitine (C3)-positive newborn screens in Wisconsin from 2013 to 2019.

	First-Tier Testing		Second-Tier Testing		Gene Name	Mutation	
	C3 (µM)	C3/C2	MMA (µM)	MCA (µM)		Allele 1	Allele 2
***Propionic Acidemia***							
1	10.08	0.44	0.16	3.39	*PCCB*	c.1606A>G	c.1606A>G
2	9.05	0.29	0.21	2.12	*PCCB*	c.1606A>G	c.1606A>G
3	6.88	0.38	0.28	3.26	*PCCB*	c.1606A>G	c.1606A>G
4	15.05	0.411	0.14	2.98	*PCCB*	c.1606A>G	c.1606A>G
5	8.34	0.28	0.04	2.48	*PCCB*	c.1606A>G	c.1606A>G
***Methylmalonic Acidemia***							
1	7.37	0.25	13.3	2.27	*MMUT*	c.1084-10A>G	c.1084-10A>G
2	8.74	0.26	13.1	1.48	*MMUT*	c.1196_1197delTG	c.2026G>A
3	9.75	0.22	2.62	0.57	*MMUT*	c.1663G>A	c.1663G>A
4	5.10	0.22	4.62	1.04	*MMUT*	c.1663G>A	c.1663G>A
5	9.39	0.32	8.87	0.76	*CD320*	c.262_264del	c.262_264del
***Cobalamin C Deficiency***							
1	15.77	0.54	70.6	5.62	*MMACHC*	c.271dupA	c.271dupA
2	9.24	0.38	35.5	3.7	*MMACHC*	c.271dupA	c.271dupA
3	20.91	0.55	84.2	6.25	*MMACHC*	c.271dupA	c.271dupA
4	11.69	0.4	48.3	2.61	*MMACHC*	c.271dupA	c.436_450del
